# Cancer Stem Cell Formation Induced and Regulated by Extracellular ATP and Stanniocalcin-1 in Human Lung Cancer Cells and Tumors

**DOI:** 10.3390/ijms232314770

**Published:** 2022-11-25

**Authors:** Jingwen Song, Yanrong Qian, Maria Evers, Corinne M. Nielsen, Xiaozhuo Chen

**Affiliations:** 1 Department of Biological Science, Ohio University, Athens, OH 45701, USA; 2 The Molecular and Cellular Biology Program, Ohio University, Athens, OH 45701, USA; 3 Edison Biotechnology Institute, Ohio University, Athens, OH 45701, USA; 4 Department of Chemistry and Biochemistry, Ohio University, Athens, OH 45701, USA; 5 The Honors Tutorial College, Ohio University, Athens, OH 45701, USA; 6 Translational Biomedical Science Program, Ohio University, Athens, OH 45701, USA; 7 Department of Biomedical Sciences, The Heritage College of Osteopathic Medicine, Ohio University, Athens, OH 45701, USA

**Keywords:** cancer metabolism, EMT, RNA sequencing, CRISPR Cas9 Knockout, metastasis, metabolic analysis, eATP, *STC1*, protein markers, animal study

## Abstract

Cancer stem cells (CSCs) are closely associated with metastasis and epithelial mesenchymal transition (EMT). We previously reported that extracellular ATP (eATP) induces and regulates EMT in cancer cells. We recently found that the gene stanniocalcin 1 (*STC1*) is significantly upregulated by eATP in human non-small lung cancer (NSCLC) A549 cells; however, the relationships among eATP, CSCs, and *STC1* were largely unknown. In this study, we performed gene knockdown and knockout, and a wide variety of functional assays to determine if and how eATP and *STC1* induce CSCs in NSCLC A549 and H1299 cells. Our data show that, in both cultured cells and tumors, eATP increased the number of CSCs in the cancer cell population and upregulated CSC-related genes and protein markers. *STC1* deletion led to drastically slower cell and tumor growth, reduced intracellular ATP levels and CSC markers, and metabolically shifted *STC1*-deficient cells from an energetic state to a quiescent state. These findings indicate that eATP induces and regulates CSCs at transcriptional, translational, and metabolic levels, and these activities are mediated through *STC1* via mitochondria-associated ATP synthesis. These novel findings offer insights into eATP-induced CSCs and identify new targets for inhibiting CSCs.

## 1. Introduction

Metastasis is associated with up to 90% of cancer mortality [1], with epithelial-mesenchymal transition (EMT) and cancer stem cells (CSC) contributing significantly to metastasis [2,3]. In addition to their metastatic involvement, CSCs play crucial roles during tumor progression [4], including self-renewal and enhanced resistance to anticancer drugs [5,6,7]. EMT is a CSC-related process that plays a vital role during the early steps of metastasis, CSC-related EMT plays a vital role by reducing cell-cell adhesion, increasing motility, responding to the tumor microenvironment (TME), in preparation for metastasis [8,9]. Secreted factors such as TGF-β and interleukins, are produced by the TME and promote EMT, CSCs, and tumor progression [8,10]. The TME also contains extraordinarily high concentrations of intratumoral extracellular ATP (eATP), at least 1000 times higher than those found in healthy tissues [11,12,13,14]. However, the functional relationships among eATP, CSCs, and EMT in cancer are not well understood.

High levels of eATP in the TME are generated from multiple sources, including necrotic and lysed tumor cells and stromal cells [15,16,17,18] and by active release of ATP from these cells [19,20,21,22,23,24]. eATP binds and activates purinergic receptors (PRs) located on the plasma membrane of normal and cancer cells, and thereby actively induces EMT [25,26,27]. Our group found that, in human non-small cell lung cancer (NSCLC) cells, eATP promotes cancer cell proliferation, migration, invasion [28,29,30], and resistance to anticancer drugs in several cancer cell lines from multiple cancer types [30,31]. We also reported that eATP induces these apparently diverse activities in cancer cells not only extracellularly, but also intracellularly both in vitro and in vivo [28,29,32], after being internalized through a process called macropinocytosis [33,34], which is one of the hallmarks of cancer metabolism [35]. As a direct result of eATP internalization, the level of intracellular ATP (iATP) is drastically elevated [28,29,32]. The cyclooxygenase 2 (COX-2)/prostaglandin E_2_ (PGE-2) inflammatory cascade also plays a role in iATP elevation [36]. Thus, iATP levels are regulated by intracellular ATP synthesis, eATP internalization, and other regulatory mechanisms [36]. Recently, we reported that eATP induces EMT in NSCLC A549 and H1299 cells by triggering cell detachment, loss of apical-basal polarity, cytoskeleton remodeling, and other key features of EMT [27]. More recently, through RNA sequencing analysis, metabolomics analyses, and functional studies, we described that eATP induces genes, proteins, and metabolites associated with EMT [37]. In the RNAseq study, we identified 11 genes [37] that are consistently and significantly upregulated by eATP and TGF-β, a well-established EMT inducer [38]. Several of the 11 genes are known to be closely involved in EMT, while other genes can be inferred to play potential roles in EMT [37].

Studying lung cancer is biomedically significant, as lung cancer is one of the most common cancer types and has the highest mortality among cancers [39]. Also, NSCLC is the largest type of lung cancer constituting about 85% of all lung cancer cases. It is further noteworthy that the lung cancer rate has been increasing among women in France for the past 20 years [40]. CSC formation in lung and other cancers is regulated by various factors including TGF-β, nuclear factor kappa-light-chain-enhancer of activated B cells (NF-kB), and the proinflammatory cytokines TNF-α/β, which trigger NF-κB [41]. Notably the TGF-β/EMT axis is critical for crosstalk within the TME leading to accumulation of CSCs [42]. Based on the studies cited above, we hypothesized that eATP induces not only EMT, but also CSCs in human NSCLC cells, and that select genes, identified from NSCLC A549 cells in the RNAseq study, are likely to be involved in mediating CSC induction and formation. To test this hypothesis, we used various bio-functional assays to study expression and functions of genes and proteins known to be involved in EMT and CSC formation. We also used in vitro and in vivo strategies, including siRNA knockdown (KD) and CRISPR-cas9 knockout (KO) to determine the contribution and mechanism of stanniocalcin-1 (*STC1*), one of the 11 genes we identified by RNAseq, during induction of CSC. *STC1* is both a secreted protein [43] and an intracellular acting protein [44], putatively working to regulate ATP synthesis in the mitochondria [45,46]. However, *STC1*′s effects on CSCs and EMT in NSCLC have not been studied.

This study demonstrates, for the first time, the inducing and regulatory activities of eATP in CSCs. It also begins to delineate mechanistic roles of *STC1* in the CSC induction process.

## 2. Results

### 2.1. eATP Increased Rates of Cell Migration and Invasion and Altered Expression of Genes Involved in CSCs

**Figure 1:**
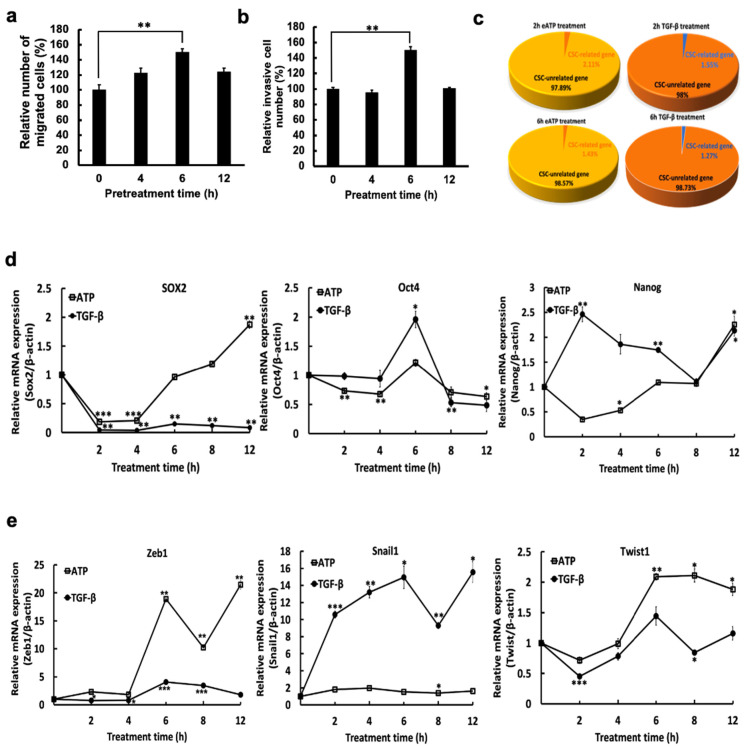
eATP induced migration, invasion, and expression of EMT- and CSC-related genes in A549 cells. Migration and invasion rates were compared between human NSCLC A549 cells, treated with 0.5 mM eATP at select timepoints, and untreated A549 cells. In addition, eATP treated cells were analyzed by RNAseq and RT-PCR, to identify changes in the expression of EMT- and CSC-related transcription factors or CSC-related genes. TGF-β (10 ng/mL) was used as a gene induction control for comparison. * *p* < 0.05, ** *p* < 0.01, and *** *p* < 0.001. (**a**) eATP treatment increased cell migration. (**b**) eATP treatment increased invasion. (**c**) Pie graphs show percentages of CSC-related genes induced by eATP or TGF-β treatment at two treatment time points in an RNAseq study. (**d**) RT-PCR of selected CSC-related transcription factor (TF) genes induced by eATP or TGF-β. (**e**) Selected EMT-related TF genes induced by eATP or TGF-β.

We first determined that 0.5 mM eATP increased both migration and invasion rates in A549 cells (Figure 1a,b). RNAseq analysis showed similar percentages of CSC-related genes induced by 0.5 mM eATP or 10 ng/mL TGF-β after 2- and 6-h treatments (Figure 1c). This result suggests that eATP and TGF-β functions similarly in induction of cancer cell motility and stimulation of CSC-associated genes.

We used qRT-PCR to show that 0.5 mM eATP induced the transcription of several well-known CSC- (Figure 1d) and EMT-related genes (Figure 1e). Over time, eATP-induced gene expression profiles were either similar to those induced by TGF-β, such as *Oct4* (Figure 1d) and *Twist1* (Figure 1e), or different from TGF-β, such as *Sox2* (Figure 1d), or *Zeb1* and *Snail1* (Figure 1e). These data indicate that eATP selectively induces expression of CSC-related genes.

Together, these results reveal that eATP induces expression of CSC-related genes in A549 cells.

### 2.2. eATP Induced Time- and Dose-Dependent Changes in Colony Formation and Levels of Proteins Involved in CSC Formation

**Figure 2:**
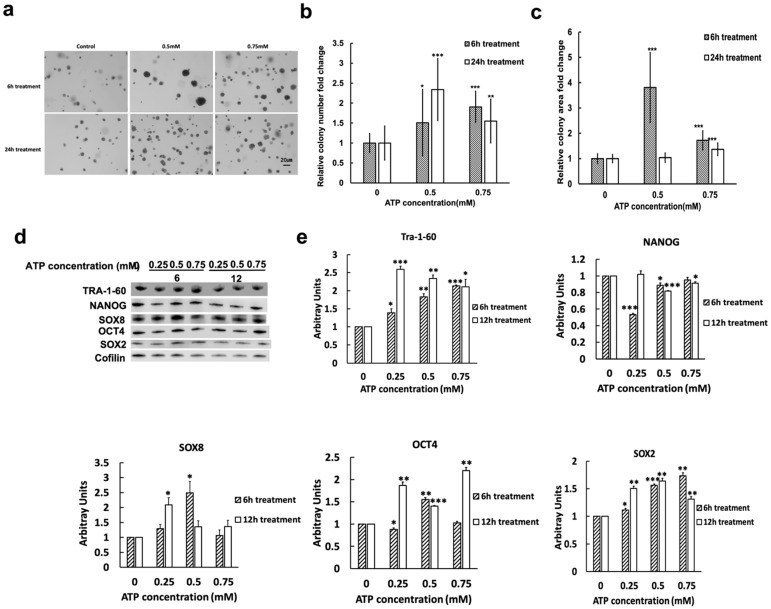
eATP induced CSC-like changes at protein, cell, and functional levels in A549 cells. A549 cells were treated with eATP at various concentrations, and then examined for colony formation and time- and dose-dependent responses in expression of CSC-related transcription factors (TFs) and CSC marker proteins. A549 cells without eATP treatment served as controls. * *p* < 0.05, ** *p* < 0.01, and *** *p* < 0.001. (**a**) Anchor-independent soft agar assay: eATP increased colony formation, confirming its CSC inducing activity. (**b**) Quantification of the soft agar assay: colony number changes in the assay (from (**a**)). (**c**) Quantification of number of colonies formed in the soft agar assay. colony size (area) changes in the soft agar assay from (**a**,**d**). Western blot analysis: eATP induced expression changes of CSC-related protein markers at different doses and different induction times (6 and 12 h). (**e**) Quantifications of eATP-induced CSC-related protein markers (from (**d**)).

Because CSCs are able to form tumor colonies [47,48,49,50], we tested whether eATP could induce anchor-independent soft agar colony formation [47] in A549 cells. We found that eATP treated A549 cells formed significantly more and larger colonies compared to untreated controls (Figure 2a–c). This data suggests that eATP exhibits CSC-promoting activity. 

Because of potential post-transcriptional and post-translational modifications, protein levels are not necessarily in parallel with their corresponding mRNA levels. Thus, we used Western blot analysis to determine which proteins were affected by eATP induction. We found that eATP induced time- and dose-dependent changes in proteins that are associated with CSCs (Figure 2d,e). Some of the protein expression changes were similar to those found for mRNA expression, while others were different. These results indicate that eATP regulates translation of select CSC-related genes in A549 cells.

These results indicate that eATP induces and mediates translational regulation for CSC-related genes in A549 cells.

### 2.3. eATP Increased CSC Surface Markers and CSC Subpopulations in NSCLC Cells

**Figure 3:**
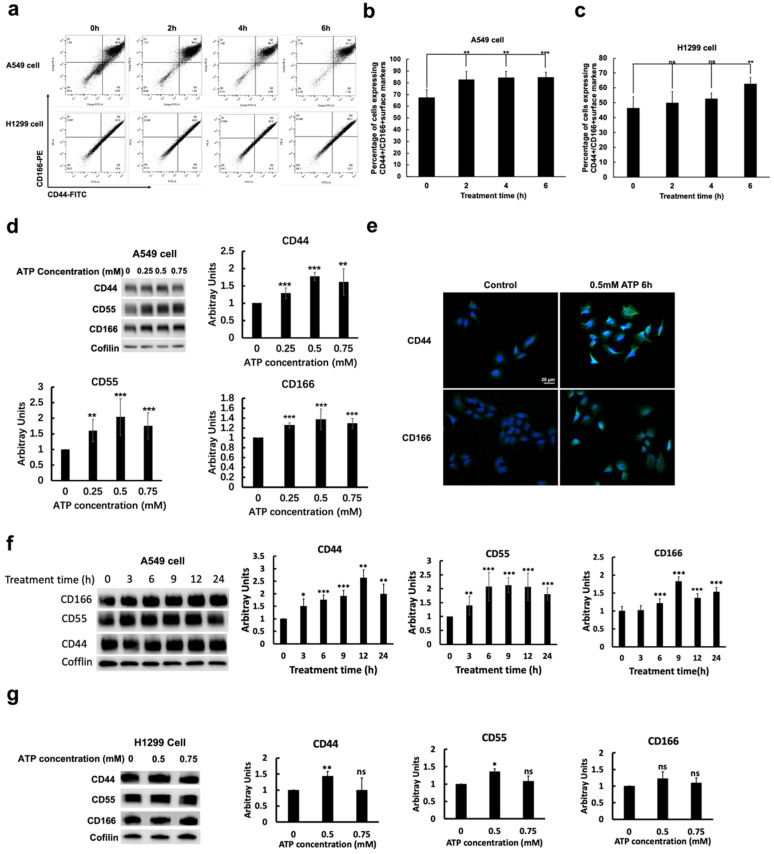
eATP induced CSC formation and CSC-related markers. Human NSCLC A549 and H1299 cells were treated with eATP at various concentrations and at different times, and then analyzed for CSC subpopulation and CSC surface protein markers. * *p* < 0.05, ** *p* < 0.01, and *** *p* < 0.001. (**a**) Cell sorter study: eATP increased the number of CD44+/CD166+ cells in the general cell population in both A549 and H1299 cells. Quantification of CSC changes in the cell sorter study for A549 cells (**b**) and for H1299 cells (**c**). (**d**) Protein analysis and quantification of CSC-related cell surface protein markers in A549 cells treated with different concentrations of eATP for 6 h. (**e**) Fluorescence microscopy of A549 cells treated with 0.5 mM eATP for 6 h. eATP-treated cells show significantly increased intensity of CSC markers CD44 and CD166 proteins. (**f**) Time-dependent expression and quantification of CSC-related cell surface protein markers in 0.5 mM eATP treated A549 cells. (**g**) Dose-dependent expression and quantification of CSC-related cell surface protein markers in H1299 cells, following 6 h eATP treatment.

Cell sorting analysis shows that eATP induced significant time-dependent increases in the number of CD44+/CD166+ cells in A549 and H1299 cells (Figure 3a). CD44, CD166, and CD55 are known CSC cell surface markers in lung cancer and other cancer types. At the final treatment time (6 h), the number of CD44+/CD166+ cells increased by approximately 15–18% for both cell lines. These results were confirmed by Western blot and immunofluorescence microscopy studies (Figure 3d,e), which were further verified by time- and dose-dependent protein analyses of CD44, CD55, and CD166 (Figure 3f,g). 

### 2.4. eATP Induced Genes Involved in EMT and CSC, and KD of STC1 Reduced CSC Phenotypic Changes

**Figure 4:**
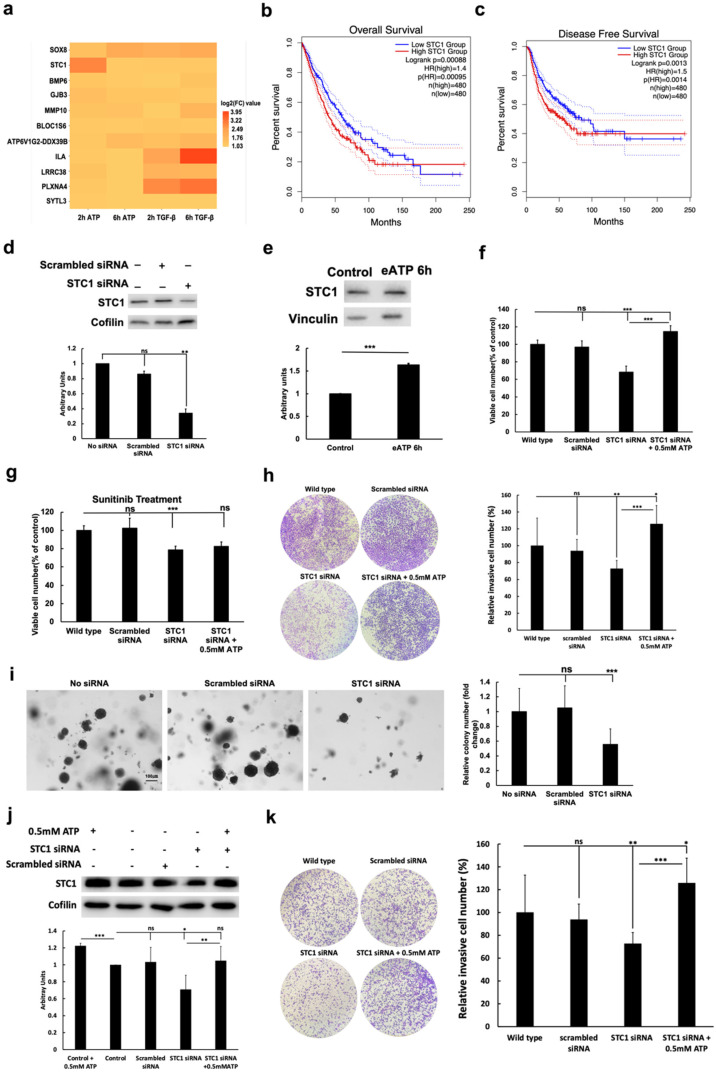
RNAseq and knockdown (KD) studies: eATP and TGF-β upregulated gene expression, and *STC1* gene knockdown led to EMT- and CSC-related changes in A549 and H1299 cells. A549 cells were treated with eATP or TGF-β for 2 or 6 h, and polyA mRNA was isolated. RNA samples were sent to a commercial service for RNAseq, and the data were analyzed. The gene *STC1*, identified from the RNAseq data, was investigated by KD studies in A549 and H1299 NSCLC cells. In these studies, untreated A549 cells served as negative controls. * *p* < 0.05, ** *p* < 0.01, and *** *p*< 0.001. (**a**) Heatmap of 11 consistently and significantly upregulated genes by both eATP and TGF-β after 2 and 6 h of treatment. (**b**,**c**) Comparison of overall survival rates (**b**) and disease-free survival rates (**c**) of high and low *STC1*-expressing human lung cancer patients. Higher *STC1* levels were associated with lower survival rate. These data were generated from GEPIA (http://gepia.cancer-pku.cn/index.html). (**d**) *STC1* gene expression was knocked down, and *STC1* protein was reduced, as analyzed by Western blot. Cofilin was used as the protein loading control. (**e**) Overexpression of *STC1* protein was induced by 0.5 mM eATP treatment for 6 h. (**f**) Viability assay of A549 cells with *STC1* gene knocked down. (**g**) Viability assay: drug resistance in A549 cells following *STC1* gene KD and 24-h treatment with target drug sunitinib (20 μM) in the presence or absence of ATP. Cell samples treated with sunitinib alone were used as controls to normalize other experimental samples in this resazurin assay. (**h**) Invasion assay: invasion of A549 cells with *STC1* gene KD. (**i**) Anchor-independent colony formation assay: Effects of *STC1* KD on the number of colonies formed; quantification of colonies. (**j**) Analysis of *STC1* protein, following siRNA KD of *STC1* in H1299 cells as examined by Western blot. Cofilin was used as the protein loading control; quantification shown in Figure S1. (**k**) Effect of *STC1* KD on the invasion of H1299 cells. Left images show transwell invasion assay; quantification shown on the right. Wildtype H1299 cells were used as controls for this assay.

In our RNAseq studies, we identified 11 genes that were consistently and significantly upregulated by both eATP and TGF-β, at both 2- and 6-h post-treatment (Figure 4a) [37]. Among these 11 genes, some are well-known for their roles in EMT and CSC formation. We also summarized the CSC-related TF genes that were up- or down-regulated by either ATP and/or TGF-β (Table 1) and CSC-related non-TF genes that were up- or down-regulated by either ATP and/or TGF-β (Table 2). Among these less studied genes, one of them is particularly noteworthy: *stanniocalcin* (*STC1*). While *STC1* has not been systematically studied, some bio-functional information for *STC1* has been reported [51,52,53,54]. In a GEPIA online service (http://gepia.cancer-pku.cn/index.html), 960 lung cancer patients were divided into high- and low-*STC1* mRNA expression groups, based on the median cutoff. As shown in Figure 4b,c, lung cancer patients with high *STC1* mRNA expression in tumor tissues had significantly worse overall survival and disease-free survival, as compared to those with low *STC1* levels (HR = 1.4, *p* < 0.001 and HR = 1.5, *p* = 0.0014, respectively). These results strongly imply the functional and clinical importance of *STC1* in lung cancer, providing rationale to focus our study on *STC1*.

To further investigate the function of *STC1* in CSCs, we specifically targeted *STC1* protein in an siRNA KD study (Figure 4d). First, we showed that *STC1* protein levels were upregulated by the treatment of eATP (Figure 4e). This result was consistent with the RNAseq study in which *STC1* mRNA was upregulated by eATP (Figure 4a). KD of *STC1* in A549 cells led to significant reductions in cell proliferation (Figure 4f), drug resistance to sunitinib (Figure 4g), invasion (Figure 4h), and colony formation (Figure 4i). Data from the KD study of *STC1* using H1299 cells (Figure 4j, Western blot; Figure 4k, invasion assay) and Figure S1 (cell viability assay) were similar to results using A549 cells (Figure 4d,h,f, respectively). These results strongly suggest the involvement of *STC1* in CSC formation and drug resistance, not only in A549 cells but also in other NSCLC cells.

### 2.5. Knockout of STC1 Lowered iATP Levels, Oxygen Consumption Rate, and Mitochondrial ATP Synthesis

**Figure 5:**
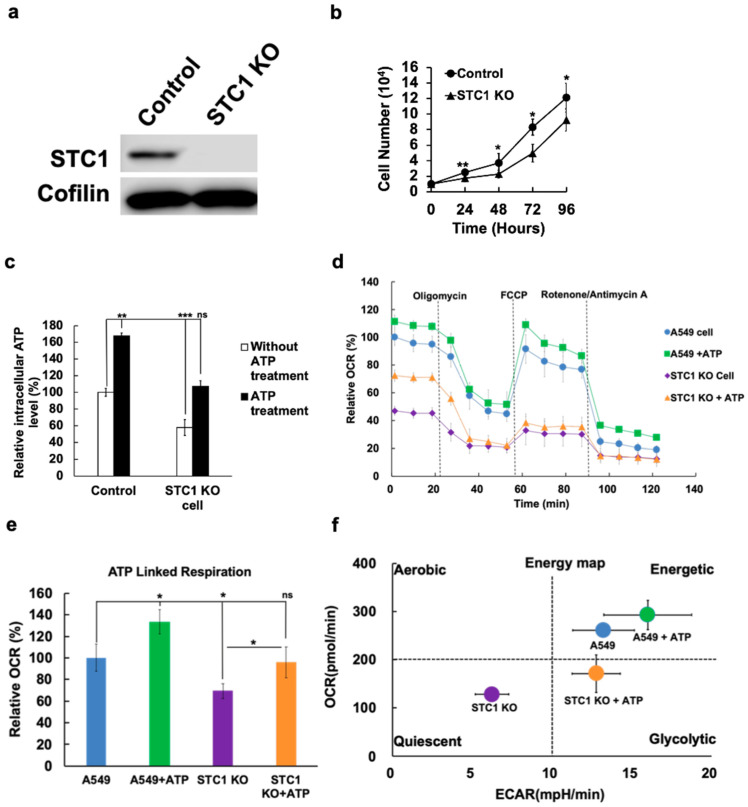
KO studies in vitro: KO of *STC1* downregulated CSC-like properties in A549 cells. The *STC1* KO in A549 cells was achieved with CRISPR-cas9 technology. KO cell clones were selected, identified, and further characterized by functional assays, in which the healthy A549 cells served as controls. * *p* < 0.05, ** *p* < 0.01, and *** *p* < 0.001. (**a**) *STC1* protein was absent from *STC1* KO (A549stc1ko) cells, as analyzed by Western blot. (**b**) KO of *STC1* resulted in slower cell proliferation rate compared with control A549 cells. (**c**) KO of *STC1* led to reduced iATP levels, and eATP treatment partially restored the iATP level. (**d**) Seahorse metabolic analysis: Mito stress test of A549 and A549stc1ko cells indicated that the relative OCR rate was reduced in A549stc1ko cells, in comparison to A549 cells. (**e**) OCR changes in the presence or absence of eATP: eATP partially restored OCR. ATP-linked respiration rate was significantly reduced in A549stc1ko cells and eATP treatment partially restored the ATP-linked respiration rate. (**f**) ECAR vs. OCR graph (energy map). eATP led to drastic changes in energetic metabolism in A549 and A549stc1ko cells as shown in an energy map. ATP treatment resulted in the left-to-right and lower-to-higher shifts of energy status for both A549 and A549stc1ko cells.

Given the *STC1* KD results, we hypothesized that knockout (KO) of the *STC1* gene would affect CSC formation through altered cell metabolism. KO of the *STC1* gene (Figure 5a) led to slower cell proliferation rate (Figure 5b) and significantly lower iATP levels, while the addition of eATP restored the iATP level to that of controls (Figure 5c). *STC1* KO also led to a relatively lower oxygen consumption rate (OCR) and ATP-linked respiration rates (Figure 5d, e). Additional analysis of Seahorse metabolic data showed that, compared with healthy A549 cells, which are in an energetic state, A549stc1ko cells were in a quiescent state (Figure 5f). When eATP was added to A549 cells, the cells became more energetic; under the same conditions, the energy status of A549stc1ko cells was converted from a quiescent (purple) to a glycolytic (orange) state (Figure 5f). Collectively, these results suggest that, as eATP is moved into cells via macropinocytosis-mediated internalization, eATP enhances the iATP pool and triggers a shift in mitochondrial energy status. This is consistent with the observation that eATP elevated the iATP level of A549stc1ko cells (Figure 5c). Thus, this study shows that *STC1* plays a major role in OXPHOS. 

Our results indicate that the roles of *STC1* in ATP synthesis and cancer metabolism are consistent with recent reports for other cancer cell types [52,53,54,55], as metastasis rates in head and neck cancer cells were decreased when iATP levels were reduced [54].

### 2.6. eATP-Treated A549 Cells Formed More Tumors at Lower Cell Injection Numbers, and Tumors Formed from A549stc1ko Cells Grew Slower and With Fewer CSCs

#### 2.6.1. First Animal Study

Since eATP induced CSC-like changes at the gene expression and protein levels several hours after treatment (particularly after 6 hours’ treatment), we further evaluated this activity in a tumor formation study, in which eATP-treated or untreated A549 cells were injected into nude mice. Our data reveal that, with fewer cells per injection, eATP-treated A549 cells, treated with 0.5 mM eATP for 6 h before injection, generated significantly more tumors than untreated-cell controls (8/10 vs. 4/10, a 100% increase in the rate, Table 3). This result suggests that in vitro eATP treatment of cells before cell injection, increased tumorigenesis, likely occurred through increased CSC numbers (Figure 3), as the CSC subpopulation is the primary factor in tumor-forming ability [47,48,49,50].

#### 2.6.2. Second Animal Study

**Figure 6:**
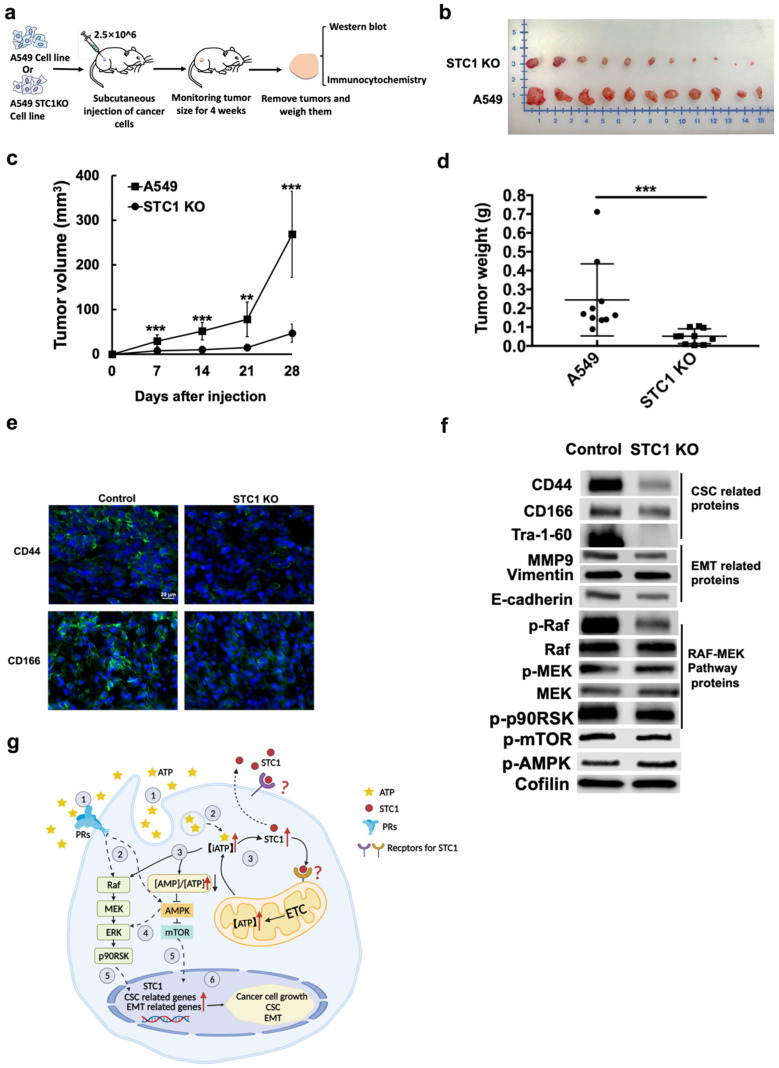
Tumor formation studies using *STC1* KO cells. A549stc1ko or A549 cells were subcutaneously injected into the flanks of nude mice to generate tumors. The tumor sizes were measured weekly for four weeks. After euthanasia, tumors were surgically removed, weighed, photographed, fixed, and then further studied by Western blot analysis and immunofluorescence microscopy. A549 tumors served as negative controls in these studies. ** *p* < 0.01, and *** *p* < 0.001. (**a**) Flow chart of the animal study. KO of *STC1* drastically reduced tumor sizes (**b**), tumor growth rate (**c**), and tumor weight (**d**). (**e**) KO of *STC1* significantly reduced expression of CSC markers CD44 and CD166 in A549stc1ko tumors compared with A549 tumors. (**f**) KO of *STC1* altered expression levels of proteins involved in EMT, CSC formation, and cell growth signaling as determined by Western blots. Quantification in Figure S2. (**g**) Hypothetical model of eATP-induced CSCs and *STC1* function in CSC formation in A549 cells. Based on our previous and current studies on eATP induced EMT and CSC in A549 and other NSCLC cells, we propose a hypothetical model for eATP’s activities. First, eATP functions as an extracellular messenger, binding and activating purinergic receptors (PRs) [27], which triggers Raf-MEK-ERK signal transduction pathway. Activated Raf-MEK-ERK signaling results in the activation of transcription factors and transcriptional changes in genes related to EMT [37] and CSC. Meanwhile, eATP is also internalized by macropinocytosis/macropinosomes and is released intracellularly to drastically increase intracellular ATP (iATP) levels [28,29]. The increased iATP in turn downregulates AMPK and upregulates mTOR, leading to accelerated cell growth. During this process, one of the upregulated genes, *STC1*, functions to upregulate mitochondrial energy metabolism, facilitate cell growth, mediate EMT, and induce CSC formation. Multiple questions remain to be answered in this model and additional studies are needed to elucidate the complete mechanisms of eATP in these processes.

The second tumor studies (Figure 6a), which were carried out with objectives independent of the first animal study, revealed that A549stc1ko tumors (at the end of the study) were approximately 75% smaller than tumors generated from A549 wildtype cells (Figure 6b), with correspondingly slower tumor growth rates (Figure 6c) and reduced tumor weights (Figure 6d). These A549stc1ko tumors were found to express much less CD44 or CD166, compared with their A549 cell controls (Figure 6e). It is also noteworthy that A549stc1ko tumor cells not only expressed lower levels of CSC markers, but also appeared to have more CD44-low and CD166-low cells. Together, these results strongly suggest that the KO of *STC1* resulted in fewer CSC cells in the tumors, consistent with our hypothesis that *STC1* participates in CSC formation and therefore tumor growth.

In the A549stc1ko tumor cells, CSC- and EMT-related proteins and phosphorylated proteins involved in the RAF- MEK signaling pathway were significantly reduced, compared with control A549 tumors (Figure 6f). In addition, phosphorylated mTOR and phosphorylated AMPK were increased and decreased, respectively (Figure 6f), indicating that mTOR/AMPK signaling is involved in iATP regulation. These data from A549stc1ko tumors are consistent with our in vitro data, which showed slower cell growth and proliferation and lower OXPHOS status (Figure 4 and Figure 5).

## 3. Discussion

Our RNAseq analysis revealed several hundred genes either upregulated or downregulated, both similarly and differentially, by eATP or TGF-β (Figure 4a, Table 1 and Table 2). These results together with the qRT-PCR data (Figure 1) demonstrate that eATP induces TGF-β-like, and EMT- and CSC-like changes at the gene expression level. Our protein expression analyses showed similar results to the gene expression studies, demonstrating that eATP induces not only EMT- but also CSC-like gene and protein expression changes (Figure 2d,e), confirming our previous findings that eATP is an EMT and potentially CSC inducer and regulator [27,37].

We speculated that some of the 11 genes identified by RNAseq could actively participate in EMT and CSC formation. Among these 11 genes, we focused on *STC1*, because of the following features. (i) *STC1* is a secreted growth factor-like glycoprotein that binds to a plasma membrane-bound receptor and functions in autocrine and paracrine manners [43]. (ii) *STC1* is also an intracellular protein [44] that binds to mitochondrial receptors, which are not fully characterized, but putatively work to increase mitochondrial oxidative phosphorylation (OXPHOS) [45,46]. (iii) *STC1* is overexpressed in most lung cancer cell lines and contributes to lung cancer cell growth [55]. ATP is well-known to be involved in EMT, and cancer cells that undergo EMT show increases/changes in intracellular ATP levels [27,37,56,57]. Thus, evidence from previous reports suggests that *STC1* plays important roles in EMT and CSC formation.

The in vitro KD and KO studies of *STC1* revealed that reduction or elimination of *STC1* expression led to reduced cell proliferation, drug resistance, cell migration and invasion, colony formation, expression of CSC markers, and CD44+/CD166+ CSC-like cells. These results suggest that *STC1* is involved in both EMT and CSC induction. While *STC1*′s multiple roles in cancer growth, metastasis, and EMT have been reported in other cancer cell lines [51,52,53,54], this is the first time that *STC1*′s role in CSC formation has been studied and described. Similar results obtained from both A549 and H1299 cells suggest that *STC1* is likely to function similarly in other human NSCLC cells. This mechanistic insight is clinically significant, as NSCLC is by far the largest subtype of lung cancer.

In vivo tumor studies showed that KO of *STC1* led to a drastically reduced tumor growth rate, significantly downregulated CSC markers (CD44 and CD166) on the surface of tumor cells, and reduced CD44+/CD166+ CSC-like cell populations within the general A549 tumor cell population. These two phenotypic changes, tumor growth rates and CSC subpopulation size, may be linked. Reduced ATP synthesis in A549stc1ko cells is likely to inhibit EMT and terminate CSC formation, thus reducing tumor growth. This is because the induction of EMT and CSCs is associated with changes in intracellular ATP levels [56,57,58]. Considering the functional roles of *STC1*, including EMT-inducing functions, which have been reported in other cancer types [51,52,53,54], it is conceivable that *STC1* has a general function in inducing EMT and CSC formation in other cancer types as well.

If *STC1* plays important roles in EMT and CSC formation, what are the mechanisms used by *STC1* in these processes? *STC1* was reported to be involved in mitochondrial ATP synthesis in human neuronal cells, most likely through upregulation of OXPHOS [45]. We showed that iATP levels were substantially reduced in *STC1* KD and KO A549 cells, suggesting that *STC1* is involved in upregulating ATP synthesis. The Seahorse metabolic analysis of A549stc1ko and regular A549 cells further implicates *STC1* in ATP synthesis (Figure 5d,e). Moreover, the KO of *STC1* from A549 cells changed their energetic state (faster energy metabolism and rapid growth) state to a quiescent state (slower energy metabolism and slower growth) in the energy map (Figure 5f). This shift was partially reversed by eATP, resulting in the two cell lines’ energetic statuses being shifted to more energetic and higher OCR states (Figure 5f). Because *STC1* is induced by both eATP and TGF-β during EMT, and because the *STC1*-induced changes in metabolic state can be reversed by eATP treatment, a mechanistic link can be established. Taken together, these data imply that *STC1* significantly contributes to CSC formation, and that mitochondrial ATP synthesis and rising iATP levels are *STC1*′s major mechanisms in EMT and CSC induction and regulation.

Based on our previous and present studies on eATP in EMT, *STC1*, and studies performed by others, we propose a hypothetical model for how eATP and *STC1* work to induce EMT and CSC in lung cancer cells (Figure 6g). Our and others’ major previous findings [25,26] support this model, as follows: eATP activates EMT by inducing cell detachment, migration and invasion, loss of apical-basal polarity, and cytoskeleton remodeling [27,37], which are key and necessary features for defining an EMT state [59,60]. eATP is also internalized by macropinocytosis to drastically increase iATP levels [27,28,29], leading to accelerated cellular energy metabolism and signal transduction through enhanced protein phosphorylation. eATP-mediated purinergic receptor signaling also significantly contributes to the process [27,61,62]. A transcriptional cofactor role for ATP in EMT and CSC induction is supported by our previous RNAseq study [37] and by this study (Figure 1, Table 1 and Table 2, and Figure 4). Involvement of Raf-MEK-ERK signaling and AMPK-mTOR signaling is supported by our protein analysis (Figure 6f). The experimental evidence from the current study further supports that eATP induces and regulates CSC formation. Moreover, *STC1* promotes OXPHOS and elevates iATP levels. Notably, a recent study reported that most lung cancer cell lines overexpressed *STC1*, and inhibition of *STC1* significantly reduced cancer cell growth [55]. Thus, eATP works both extracellularly and intracellularly, as a messenger, a mediator of energetic state and signaling, and a transcriptional co-factor to activate expression of *STC1* and other genes to induce EMT and CSC formation. Additional studies are required for final validation of this model.

## 4. Materials and Methods

### 4.1. Cell Lines and Cell Culture

Human NSCLC A549 and H1299 cell lines were purchased from American Type Culture Collection (ATCC, VA, USA). A549 and H1299 cells were cultured in standard Dulbecco’s Modified Eagle Medium with 25 mM glucose supplemented with 10% fetal bovine serum, 1% penicillin, and 50 μg/mL streptomycin. Cells were grown in a 5% CO_2_ and 37 °C incubator.

### 4.2. Transwell Migration and Invasion Assays

Cellular migration and invasion capacities were determined in 24-well transwell plates with A549 and H1299 cells as described previously [26,35]. The numbers of migrated and invaded cells were counted and averaged from six randomly selected visual fields using compound light microscopy (200× magnification).

### 4.3. qRT-PCR

Total RNA was extracted using GeneJET RNA Purification Kit (Thermo Fisher Scientific™, Waltham, MA, USA) following manufacturer’s instruction. 1 μg of RNA was reverse transcribed into cDNA using Maxima First Strand cDNA Synthesis Kit for qRT-PCR (Thermo Fisher Scientific™). qRT-PCR was carried out following the Maxima SYBR Green/Fluorescein qPCR Master Mix (Thermo Scientific™) protocol. The PCR setting were as follows: 5 min at 95 °C; 40 cycles of amplification at 95 °C for 15 s; 58 °C for 20 s; and 72 °C for 30 s. The primers amplifying *SOX2*, *NANOG*, *OCT4*, *TWIST1*, *ZEB1*, and *SNAI1* are listed in Supplemental Table S1. Gene expression levels were normalized by β-actin and quantified using the 2^−ΔΔCt^ method.

### 4.4. Colony Formation Assay

The ability of eATP to induce CSC-like anchorage-independent colonies was assessed according to a published protocol [41]. A549 cells, pretreated with or without ATP for 6 or 12 h, were seeded in a 0.3% cell agar base layer, which was on top of a 0.6% base agar layer in 6-well culture plates. 0.3% cell agar feeder layers were added weekly until colonies were observed, approximately 15 days. Colonies were counted from ten representative visual fields per condition in experimental triplicate, then averaged, using Biotek Cytation 3 (Biotek, Winooski, VT, USA). The colony areas were measured using ImageJ software (Version 1.x, Public Domain license BSD-2, National Institutes of Health (NIH), Bethesda, MD, USA).

### 4.5. Western Blot Analysis

Protein isolation and western blot analysis were performed as previously described [26,37]. Briefly, protein was isolated by sonication of treated cancer cells or tumors in lysis buffer, followed by centrifugation. Protein concentration in isolated supernatant was quantified by a bicinchoninic acid assay (BCA) protein measurement method. Protein signals were detected by Super Signal West Pico Chemiluminescent substrate (Thermo Fisher Scientific) and were developed using Odyssey Fc 2800 (LI-COR Biosciences, Lincoln, NE, USA). Software ImageJ (NIH) was used quantify protein bands in Western blots. Cofilin was used as an internal protein loading control for normalizing protein signals, as cofilin’s levels were more stable than those of β-actin, under the eATP treatment. 

### 4.6. Immunofluorescence Microscopy

Immunofluorescence was performed based on the manufacturer’s instruction. Briefly, A549 cell were seeded in 96-well plates with 0.5 mM ATP for 6 h and then fixed in 4% paraformaldehyde for 15 min at room temperature. The cells were rinsed three times with 1X PBS for 5 min each, then incubated with blocking buffer (3% Triton X-100 and 5% goat serum in PBS) for 1 h. After washing with PBS, cells were incubated with primary antibodies (CD44 (Rabbit, 1:50, Abcam, Cambridge, MA, USA, ab157107), CD166 (Rabbit, 1:50, Abcam, ab109215) at 4 °C overnight. Then, cells were rinsed three times in 1X PBS for 5 min each, followed by incubating cells with Alexa 488-conjugated goat anti-rabbit IgG secondary antibody for 1–2 h in the dark. After PBS washing, the cells were stained with Prolong^®^ Gold Antifade Reagent with DAPI (Thermo Fisher Scientific) for visualizing cell nuclei. Finally, images were taken using a Fluor Motorized DIC Polarization Phase Contrast Microscope (Zeiss AXIO Observer) at 400× magnification.

### 4.7. Flow Cytometry/Cell Sorter Assay

To analyze CSC surface markers, 1×106 A549 or H1299 cells, treated for various times with or without 0.5 mM ATP, were dissociated into a single-cell suspension. The suspended cells were incubated with antibodies CD44-FITC (555478; Isotype: Mouse IgG2b, κ), CD166-PE (559263; Isotype: Mouse IgG1, κ) (BD BioSciences, San Jose, CA, USA) at 4 °C in the dark for 30 min, followed by washing with PBS/2% FBS. Each cell pellet was resuspended in 300–500 μL PBS/2% FBS and filtered through a 40 μm cell strainer to obtain a single-cell suspension before sorting. The expression of CSC markers (CD166, CD44) was analyzed. and populations of cells expressing different markers were separated using a fluorescence-activated cell sorter (FACSAria III, BD Biosciences). The sorting for each cell population was repeated in three independent experiments and averaged.

### 4.8. RNA Sequencing

RNAseq was performed as previously described [35]. Data was stored in the Gene Expression Omnibus (GEO) database under Accession Number: GSE160671. The differentially expressed genes (DEGs) were selected with log_2_ (fold change) ≥ 1 or log_2_ (fold change) ≤ −1, and with *p* values < 0.05. The survival analysis of *STC1* gene for lung cancer patients were performed in Gene Expression Profiling Interactive Analysis web service (GEPIA, http://gepia.cancer-pku.cn/index.html) and log-rank *p* value < 0.05 was considered significant. Overall survival (OS) and disease-free survival (DFS) of the patients were analyzed.

### 4.9. Cell Proliferation and ATP Assays

Cell viability/proliferation assays were performed using resazurin dye as previously described [27,37]. A plate reader was used for spectrophotometric measurements, in order to quantify data. Intracellular ATP levels were measured by a firefly luciferase based ATPlite Luminescence assay system (PerkinElmer, WALTHAM, MA, USA) according to manufacturer’s instructions. Relative intracellular ATP levels were normalized to the control group.

### 4.10. *STC1* Gene Knockdown (KD) and Knockout (KO)

The KD small interference RNA (siRNA) for *STC1* and negative control (scrambled) siRNA were purchased from Qiagen (Germantown, MD, USA). The target sequence for *STC1* siRNA is 5′-CTGCTTAAACAAAGCAGTATA-3′. siRNA transfection was performed according to the manufacturer’s instruction and as previously described [29], using Lipofectamine RNAiMAX transfection reagent (Thermo Fisher Scientific). The KD efficiency was determined by western blot using an anti-*STC1* antibody (Abcam). The KO of *STC1* gene was performed as described previously [26]. The CRISPR gRNA containing 20-nucleotide target sequences of *STC1* (3′ACCCACGAGCTGACTTCAAC-5′) was designed and prepared by GenScript (Piscataway, NJ, USA). Homogeneous cell populations (double allele KO cells) were selected for subsequent studies.

### 4.11. Cell Growth Curve

A549 and *STC1* gene knock out A549 (A549stc1ko) cells were seeded in 24-well plates with similar cell number/density. Cell numbers were visualized under a microscope and manually counted every 24 h for four days. For each cell type, at least four randomly chosen visual fields were counted and averaged each day.

### 4.12. Seahorse Metabolic Studies

Metabolic analysis was performed using an XFe 24 Extracellular Flux Analyzer (Seahorse Bioscience, Santa Clara, CA, USA). Briefly, mitochondrial OXPHOS related oxygen consumption rate (OCR) or glycolysis-related extracellular acidification rate (ECAR) of 60,000 A549 or A549stc1ko cells, treated with or without eATP (0.5 mM), was measured continuously with the analyzer using a Seahorse XF Cell Mito Stress Test Kit (Agilent, Santa Clara, CA, USA) according to manufacturer’s instructions and as previously described [63].

### 4.13. Tumor Studies

Five-week-old male nude mice of Nu/Nu strain were purchased from the Jackson Laboratory (Bar Harbor, ME, USA) and fed irradiated Teklad Global rodent diet with 19% protein from Harlan Laboratories. In the tumor number study, A549 cells, pretreated with or without 0.5 mM ATP for 6 h, were then subcutaneously injected into the flank of nude mice at 3 × 10^6^, 1 × 10^6^, 3 × 10^5^, and 1 × 10^5^ cells per injection. Injections of A549 cells without ATP treatment served as controls. N = 10 per group. One-month post-injection, mice were euthanized, and tumors were surgically removed and counted. The minimal cell numbers required for tumor formation were compared between the ATP pretreatment groups and the no-ATP treatment control group.

In the animal study with KO cells, A549 or *STC1* gene knock out A549 (A549stc1ko) cells were injected subcutaneously into the flank of nude mice at 5×10^6^ cells per injection. Mice fed irradiated Teklad Global rodent diet with 19% protein from Harlan Laboratories. N = 11 mice per group. Tumor size was measured using digital calipers twice a week, and tumor volume was calculated as (length × width × width)/2 in mm^3^. One month after cell injection, mice were euthanized and their tumors were surgically removed, weighed, and photographed. Approximately one third of each tumor was used for protein isolation and Western blots, and one third of each tumor was used for immunofluorescence studies.

### 4.14. Immunocytochemistry Study of Tumor Sections

Frozen tumors from the animal studies were cryopreserved in 30% sucrose solution and then embedded in OCT (Tissue-Tek, Torrance, CA, USA). 12 μm thick tumor sections (Leica CM1950 Cryostat, Leica Biosystems, Deer Park, IL, USA) were fixed in 4% paraformaldehyde (PFA) and incubated in goat serum buffer for 1 h. After washing with PBS, primary antibodies were: CD44 (Rabbit, 1:50, Abcam, ab157107), and CD166 (Rabbit, 1:50, Abcam, ab109215), and secondary antibody was goat anti-rabbit-Alexa Fluor488 (1:1000, Cell signaling, Danvers, MA, USA). Sections were mounted with ProLong Gold Antifade Mountant with DAPI (Thermo Fisher Scientific). Images were acquired using a Fluor Motorized DIC Polarization Phase Contrast Microscope (Zeiss AXIO Observer) with consistent intensity and exposure parameters at 400× magnification.

### 4.15. Statistical Analysis

Each experiment was performed in at least triplicate, and all experiments were repeated at least once. Results were presented as mean ± standard deviation. Student’s *t*-test was performed to evaluate the differences between two groups, and nonparametric ANOVA was used to evaluate differences among multiple groups. *p* < 0.05 was considered statistically significant. * *p* < 0.05, ** *p*< 0.01 and *** *p* < 0.001.

## 5. Conclusions

In summary, our in vitro and in vivo data demonstrate that eATP and *STC1* induce and regulate CSCs at multiple levels during tumorigenesis. During CSC induction and formation, eATP performs the initiating and inducing functions. Then, eATP-induced *STC1* plays some vital stimulatory and regulatory roles. One of *STC1*′s mechanisms in CSC induction is through the upregulation of OXPHOS and iATP levels. These new findings justify further studies of eATP, *STC1* and the ten other eATP-induced genes for their functions in EMT induction and CSC formation. These future studies include exactly how *STC1* upregulates ATP synthesis and *STC1*′s functional relationships with other EMT- and CSC-inducing genes/proteins. Our identification of *STC1* and ten other genes consistently upregulated by eATP [37] also provides novel candidate anticancer targets for inhibiting signaling via ATP and thus slowing down and/or blocking EMT, CSC formation, and metastasis [64]. 

## Figures and Tables

**Table 1:** CSC-related transcription factor genes up- or down-regulated by either ATP and/or TGF-β.

		Log2(FC) Values			
Gene Symbol	Gene Name	ATP 2h	TGF-β 2h	ATP 6h	TGF-β 6h
1. *FoxO1*	Forkhead box O1	**2.36**	**1.23**	0.69	**1.07**
2. *FOSL1*	FOS like 1, AP-1 transcription factor subunit	**2.44**	0.58	**1.24**	0.14
3. *FOS*	Fos proto-oncogene, AP-1 transcription factor subunit	**−** **4.20**	**−3.21**	**−1.05**	**−** **3.32**
4. *JUNB*	JunB proto-oncogene, AP-1 transcription factor subunit	**−** **1.34**	**2.30**	−0.16	**2.34**
5. *JUN*	Jun proto-oncogene, AP-1 transcription factor subunit	0.50	**1.20**	0.05	**1.34**
6. *c-Maf/maf*	MAF bZIP transcription factor	0.81	**3.54**	0.80	**4.75**
7. *MITF*	Melanocyte inducing transcription factor	**1.10**	−1.00	0.16	**−** **1.16**
8. *NFkB1*	Nuclear factor kappa B subunit 1	**1.49**	**1.13**	0.05	0.97
9. *SNAI1/Snail 1*	Snail family transcriptional repressor 1	0.21	**2.85**	0.50	**3.13**
10. *SOX8*	SRY-box 8	**2.01**	**2.72**	**2.94**	**2.91**
11. *SOX4*	SRY-box 4	0.62	**1.28**	0.10	0.93
12. *SOX21*	SRY-box 21	−0.51	−**2.00**	0.28	**−** **2.91**
13. *SOX2*	SRY-box 2	−**1.90**	−**2.90**	−0.73	**−** **3.39**
14. *Oct-3/4—POU5F1*	POU class 5 homeobox 1	0.31	0.85	0.76	0.94
15. *Nanog*	Nanog homeobox	−4.92	2.83	−4.92	−4.92

Note: Log2(FC) values for untreated controls were assigned as 0 for comparison. Log2(FC) values above 0 represent upregulation, while values below 0 represent downregulation. The bold-faced values in the table were significantly different from the untreated control = 0.

**Table 2:** CSC-related non-TF genes up- or down-regulated by either ATP and/or TGF-β.

		Log2(FC) Values			
Gene Symbol	Gene Name	ATP 2h	TGF-β 2h	ATP 6h	TGF-β 6h
1. *BMP7*	Bone morphogenetic protein 7	−0.09	**1.36**	−0.66	0.69
2. *E-Cadherin/CDH1*	Cadherin 1	0.23	0.04	−0.82	**−1.17**
3. *LMO2*	LIM domain only 2	0.99	**1.20**	0.96	0.56
4. *NOTCH1*	Notch 1	**−1.45**	0.24	0.28	0.17
5. *Sonic Hedgehog/SHH*	Sonic hedgehog signaling molecule	−0.29	**1.51**	−0.76	0.38
6. *TRA-1-81/PODXL/TRA-1-60*	Podocalyxin like	−0.16	0.26	0.62	**1.99**
7. *Vimentin/VIM*	Vimentin	−0.10	0.36	0.18	**1.06**
8. *CXCR4*	C-X-C motif chemokine receptor 4	**−1.19**	**−1.02**	−0.21	−0.87
9. *IL6R*	Interleukin 6 receptor	−0.10	−0.23	**1.12**	**−2.30**
10. *Aminopeptidase N/CD13/ANPEP*	Alanyl aminopeptidase, membrane	0.35	0.58	0.42	**1.09**
11. *CXCL8/IL-8*	C-X-C motif chemokine ligand 8	**1.93**	0.73	0.56	0.28
12. *IL6*	Interleukin 6	**2.10**	0.99	0.60	**2.08**

Note: Log2(FC) values for untreated control were assigned as 0 for comparison. Log2(FC) values above 0 represent upregulation while values below 0 represent downregulation. The bold-faced values in the table were significantly different from the untreated control = 0.

**Table 3:** Numbers of tumors generated from injecting different number of ATP-pretreated or untreated A549 cells.

Number of Tumors Generated out of 10 Injections (X/10)		
Number of Cells Injected	ATP Pretreatment	Without ATP Pretreatment
**3 × 10^6^**	**10/10**	**10/10**
**1 × 10^6^**	**10/10**	**9/10**
**3 × 10^5^**	**9/10** 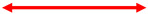 **8/10**	
**1 × 10^5^**	**8/10** 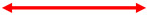 **4/10**	

Note: A549 cells were incubated with 0.5 mM of ATP for 6 h before injecting into the flank of nude mice.

## Data Availability

RNA sequencing data can be accessed via the Gene Expression Omnibus (GEO) database, Accession Number GSE160671.

## Supplementary Materials

The following are available online at https://www.mdpi.com/article/10.3390/ijms232314770/s1, Figure S1. *STC1* KD studies in H1299 cells; Figure S2. Quantification of western blots shown in Figure 6f; Table S1. Real-time (RT) PCR primers; Table S2. Antibody information list of western blot analysis.
