# Supplementary material for: Cancer Stem Cell Formation Induced and Regulated by Extracellular ATP and Stanniocalcin-1 in Human Lung Cancer Cells and Tumors
Source: Int J Mol Sci. 2022 Nov 25;23(23):14770. doi: 10.3390/ijms232314770 (PMC9740946; doi:10.3390/ijms232314770)
Supplement: Supplementary file 1 [file ijms-23-14770-s001.zip › ijms-1983122-supplementary.pdf]

International Journal of
Molecular Sciences

MDPI

Supplementary

Cancer Stem Cell Formation Induced and Regulated by Extracellular ATP and Stanniocalcin-1 in Human Lung Cancer Cells and Tumors

Jingwen Song[1,2,3], Yanrong Qian[2,3,4] and Maria Evers[3,5] Corinne M. Nielsen[1,2,6] Xiaozhuo Chen[1,2,3,4,7,*]

1 Department of Biological Science, Ohio University, Athens, OH 45701, USA
2 The Molecular and Cellular Biology Program, Ohio University, Athens, OH 45701, USA
3 Edison Biotechnology Institute, Ohio University, Athens, OH 45701, USA
4 Department of Chemistry and Biochemistry, Ohio University, Athens, OH 45701, USA
5 The Honors Tutorial College, Ohio University, Athens, OH 45701, USA
6 Translational Biomedical Science Program, Ohio University, Athens, OH 45701, USA
7 Department of Biomedical Sciences, The Heritage College of Osteopathic Medicine, Ohio University, Athens, OH 45701, USA
* Correspondence: chenx@ohio.edu

Citation: Song, J.; Qian, Y.; Evers, M.; Nielsen, C.M.; Chen, X. Cancer Stem Cell Formation Induced and Regulated by Extracellular ATP and Stanniocalcin-1 inHuman Lung Cancer Cells and Tumors. *Int. J. Mol. Sci.* **2022**, *23*, 14770. https://doi.org/10.3390/10.3390/ijms232314770

Academic Editor: Nam Deuk Kim

Received: 6 October 2022
Accepted: 15 November 2022
Published: 25 November 2022

Publisher's Note: MDPI stays neutral with regard to jurisdictional claims in published maps and institutional affiliations.

Copyright: © 2022 by the authors. Submitted for possible open access publication under the terms and conditions of the Creative Commons Attribution (CC BY) license (https://creativecommons.org/licenses/by/4.0/).

FigureS1

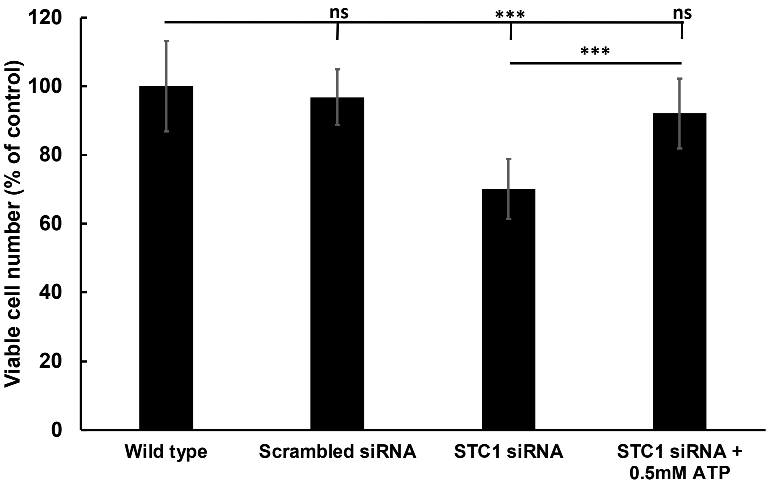

Figure S1. STC1 KD studies in H1299 cells. Cell viability / proliferation assay of H1299 cells with STC1 gene knocked down. *** p<0.001, ns = not significant. This figure is related to regular Figure 4 (KD study).

FigureS2

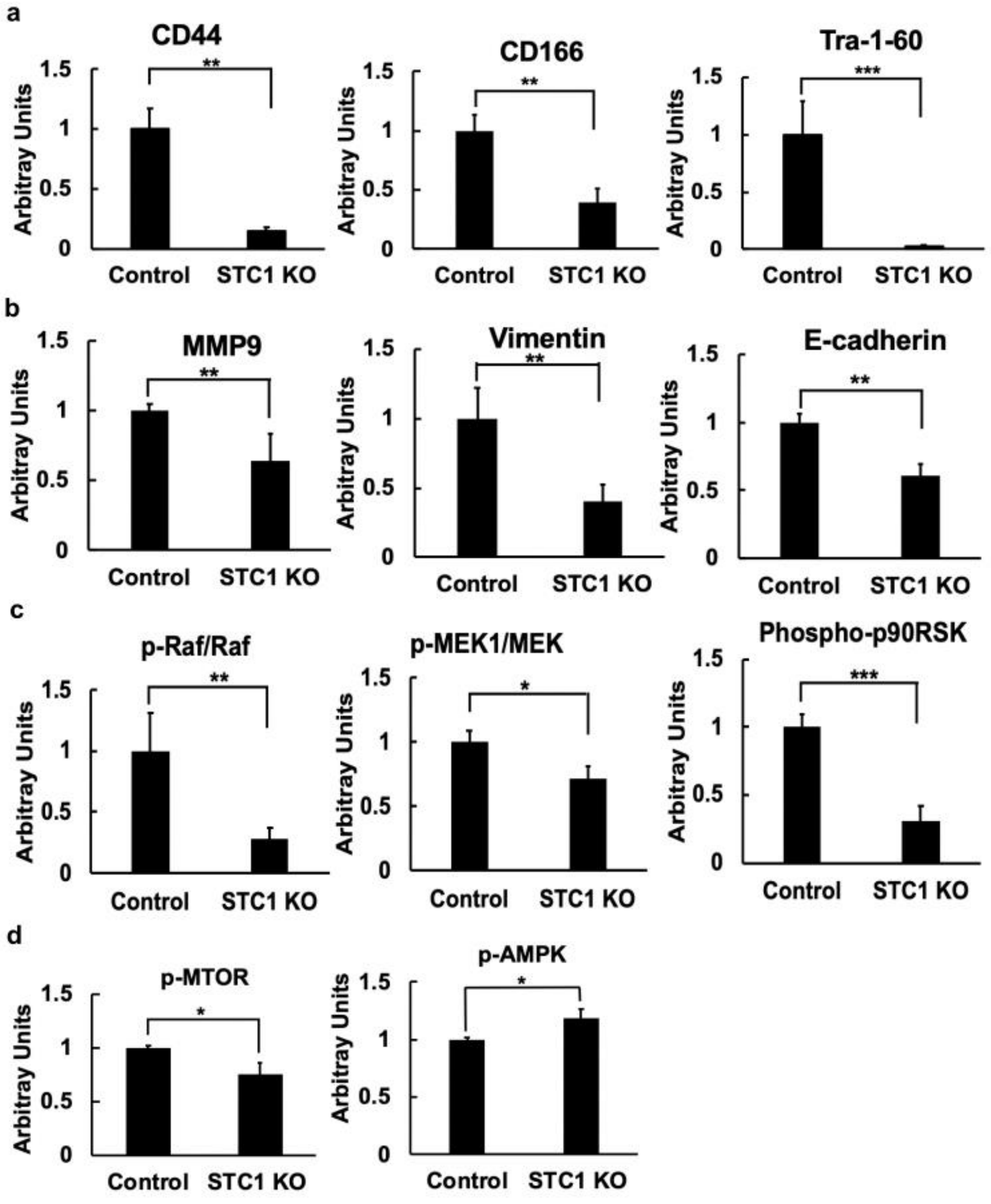

Figure S2. Quantification of western blots shown in Fig 6f. Quantification of protein expression levels after KO of *STC1* genes involved in CSC formation. **(a).** CSC-related markers. **(b).** EMT-related markers. (**c**). Phosphorlayed protein markers in the Raf-MEK signaling pathway. **(d)**. Phosphorylated

mTOR and AMPK, proteins involved in ATP synthesis. *p<0.05, **p<0.01, ***p<0.001.

Table S1. Real-time (RT) PCR primers.

Gene	Primer sequence
SOX2	Forward:5′-ATAATAACAATCATCGGCGG-3′
	Reverse: 5′-AAAAAGAGAGAGGCAAACTG-3'
NANOG	Forward: 5′-CCAGAACCAGAGAATGAAATC-3′
	Reverse: 5′-TGGTGGTAGGAAGAGTAAAG-3'
OCT4	Forward: 5′-GATCACCCTGGGATATACAC-3′
	Reverse: 5′-GCTTTGCATATCTCCTGAAG-3'
TWIST1	Forward: 5′-CTAGATGTCATTGTTTCCAGAG- 3′
	Reverse: 5′-CCCTGTTTCTTTGAATTTGG-3'
ZEB1	Forward: 5′-AAAGATGATGAATGCGAGTC-3′
	Reverse: 5′-TCCATTTTCATCATGACCAC-3'
SNAI1	Forward: 5′-CTCTAATCCAGAGTTTACCTTC-3′
	Reverse: 5′-GACAGAGTCCCAGATGAG-3'
**β-*actin***	Forward: 5′-GACGACATGGAGAAAATCTG-3′
	Reverse: 5′-ATGATCTGGGTCATCTTCTC-3′

Supplemental Table S2. Antibody information list of western blot analysis

Antibody	Antibody dilution	Product information	Monoclonal type
SOX2	1:1000	Abcam, ab97959	Rabbit
SOX8	1:1000	Abcam, ab104245	Rabbit
OCT4	1:1000	Abcam, ab19857	Rabbit
Nanog	1:1000	Abcam, ab21624	Rabbit
TRA-1-60	1:1000	Abcam, ab16288	Mouse
CD44	1:1000	Abcam, ab157107	Rabbit
CD166	1:2000	Abcam, ab109215	Rabbit
CD55	1:2000	Abcam, ab133684	Rabbit
STC1	1:2000	Abcam, ab229477	Rabbit
Raf-1	1:1000	Santa Cruz,sc-133	Rabbit
Anti-p-Raf-1	1:1000	Santa Cruz,sc-271929	Rabbit
Anti-p-MEK-1/2	1:1000	Santa Cruz,sc-7995	Rabbit
MEK-1/2	1:1000	Santa Cruz,sc-436	Rabbit
Phospho-p90RSK	1:1000	Cell Signaling,11989	Rabbit
MMP9	1:1000	Cell Signaling,2270	Rabbit
Vimentin	1:1000	Cell Signaling,5741	Rabbit
E-cadherin	1:1000	Cell Signaling,3195	Rabbit
Phospho-mTOR	1:1000	Cell Signaling,5536	Rabbit
Phospho-AMPKα	1:1000	Cell Signaling,2535	Rabbit
Anti-rabbit Secondary antibody	1:2000	CST, # 7074	Rabbit
Vinculin	1:3000	Cell signaling, 4650	Rabbit
Cofilin (D3F9) XP®	1:3000	Cell signaling, 5175	Rabbit
